# EEG microstate reconfiguration across cognitive states in preschool children with developmental language disorder

**DOI:** 10.3389/fnins.2026.1844934

**Published:** 2026-08-17

**Authors:** Aimin Liang, Hanxiao Wang, Yang Shi, Chunyan Qu, Zhuang Wei, Zhijun Cui, Xu Zhang, Xin Ni, Xiaolin Ning, Jiancheng Fang

**Affiliations:** 1 Children’s Health Care Center, Beijing Children’s Hospital, Capital Medical University, National Center for Children’s Health, Beijing, China; 2 School of Instrumentation Science and Optoelectronic Engineering, Beihang University, Beijing, China; 3 Beijing Children’s Hospital, Capital Medical University, National Center for Children’s Health, Beijing, China

**Keywords:** cognitive context, developmental language disorder, EEG microstates, microstate switching organization, preschool children

## Abstract

**Introduction:**

Developmental language disorder (DLD) has been associated with atypical neural processing, but it remains unclear whether preschool children with DLD show altered EEG microstate organization across cognitive states. Most microstate studies in clinical populations have focused on resting-state activity, and much less is known about how microstate profiles vary across task contexts in young children.

**Methods:**

EEG was recorded from preschool children with DLD (*n* = 12) and typically developing (TD) children (*n* = 22) during three consecutive paradigms: a video-based rest-like viewing condition (Rest), semantic picture–sound matching (Match), and passive auditory oddball listening (Oddball). Within-condition template extraction and cross-condition alignment were used to quantify conventional microstate features, including mean duration, time coverage, occurrence rate, and global explained variance (GEV). Directed transition probabilities and condition-difference Δ-transition indices were additionally summarized as exploratory descriptors of microstate switching organization. Conventional microstate features were analyzed using mixed-design repeated-measures ANOVA.

**Results:**

Multiple conventional microstate features differed across Rest, Match, and Oddball, indicating that EEG microstate profiles varied across task contexts under the condition-specific template framework. In contrast, Group main effects and Group × Condition interactions did not provide robust evidence of DLD-TD differences in conventional microstate features. Full transition matrices also showed descriptive condition-related variation in switching organization, whereas group-related deviations in transition probabilities were generally small. Among the representative exploratory Δ-transition pathways, one Oddball-Match pathway showed a nominal uncorrected group difference, but this effect did not survive full-family false discovery rate correction.

**Discussion:**

EEG microstate profiles in preschool children appear sensitive to task context, whereas evidence for DLD-specific microstate alterations was limited in the present sample. Because templates were extracted separately within each condition and aligned post hoc, the condition-related findings should be interpreted as descriptive evidence of task-context-related microstate organization rather than as a strict common-template test of condition effects. Larger, better-characterized samples and common-template approaches are needed to clarify whether reproducible DLD-related alterations in EEG microstate organization are present across cognitive contexts.

## 1 Introduction

Developmental language disorder (DLD) is a common neurodevelopmental condition characterized by persistent difficulties in language acquisition and use that cannot be adequately explained by intellectual disability, hearing loss, neurological damage, insufficient language exposure, or other biomedical or environmental factors (Bishop et al., 2017). Although its behavioral manifestations have been well documented, the large-scale neural dynamics associated with DLD remain insufficiently understood, particularly in preschool children. This gap is important because early childhood is marked by rapid brain network development and reorganization, and subtle alterations in neural coordination may not be fully captured by behavioral measures alone. Electroencephalography (EEG), with its high temporal resolution and feasibility in young children, provides a suitable approach for examining these fast neural processes. Because DLD emerges early and shows substantial heterogeneity, objective neurophysiological measures may be relevant for early screening, subgroup characterization, and intervention monitoring (Yavuz et al., 2026). Recent EEG studies in children with DLD continue to indicate atypical neural signatures at both resting-state and task-evoked levels, including altered speech perception, mismatch responses, cortical tracking of speech, and baseline oscillatory organization, although the precise form of these abnormalities remains heterogeneous (Campos et al., 2024, 2025; Liang et al., 2026; Nora et al., 2024; Stanojevic et al., 2023). Against this background, it is important to determine whether atypicality in preschool DLD is expressed not only in isolated EEG markers but also in the large-scale temporal organization of brain states. This question is further motivated by earlier work showing that atypical auditory discrimination and mismatch-related responses are repeatedly implicated in developmental language and literacy difficulties (Bishop, 2007; Kujala and Leminen, 2017).

EEG microstate analysis provides a temporospatial framework for describing continuous EEG as a sequence of brief, quasi-stable scalp topographies (Pascual-Marqui et al., 1995; Khanna et al., 2015; Michel et al., 2024). Microstates typically remain stable for tens to hundreds of milliseconds before rapidly transitioning to another state, offering a compact description of ongoing brain dynamics. Early work showed that temporospatial EEG characteristics are sensitive to cognitive dysfunction, with impaired cognition associated with altered microstate duration and fragmentation of stable topographic patterns (Stevens and Kircher, 1998). More recent studies have shown that microstate abnormalities are not limited to conventional temporal descriptors such as duration and coverage but may also be reflected in the organization, complexity, and predictability of state transitions (Tait et al., 2020; Wu et al., 2025). Together, these findings suggest that EEG microstates may offer a useful multilevel framework for studying atypical neural dynamics in developmental disorders, a view supported by recent reviews summarizing their clinical application across psychiatric and neurodevelopmental conditions (Casas Osorio et al., 2025; Das et al., 2022; Vass et al., 2025). Normative developmental changes in EEG microstates have also been described across childhood and adolescence, further supporting the relevance of microstate analysis for developmental populations (Koenig et al., 2002). Importantly, EEG microstates describe large-scale spatiotemporal dynamics at the scalp level, and their relationships with specific cognitive functions, language networks, or neural generators remain indirect; functional interpretations should therefore be made with appropriate caution (Michel et al., 2024; Tarailis et al., 2024).

Most existing microstate studies, however, have been conducted in adult clinical populations and have focused primarily on resting-state activity. In cognitive impairment and Alzheimer’s disease, for example, altered microstate duration, coverage, and transition complexity have been linked to disease-related neural dysfunction and clinical progression (Tait et al., 2020; Wu et al., 2025). These findings are informative because they show that both conventional temporal parameters and higher-order dynamic descriptors can capture clinically meaningful changes in brain function. Even so, such conclusions cannot be directly generalized to preschool DLD, which differs substantially from neurodegenerative disorders in developmental timing, symptom profile, and expected neural phenotype. In addition, relatively few studies have examined whether microstate organization in young children varies across different cognitive contexts, such as passive rest-like viewing, semantic matching, and auditory monitoring. More broadly, recent reviews of developmental EEG markers continue to emphasize both the promise of early electrophysiological biomarkers and the methodological heterogeneity of this literature in preschool populations (Christodoulou et al., 2026). Related work in toddlers with receptive or expressive language delay further suggests that developmental EEG atypicality may extend beyond task-evoked responses to sleep-related neural organization (Hong et al., 2025).

This question is particularly relevant in preschool DLD, where neural differences may be subtle and may not appear as large group effects in the average temporal expression of individual microstates. Instead, atypicality may be reflected in how brain-state organization varies across task contexts or in how the system transitions from one state to another. Conventional microstate features and transition-based measures therefore capture related but distinct aspects of ongoing brain activity. Examining both may help clarify whether DLD-related differences are better characterized by changes in state-wise temporal expression or by changes in switching organization.

To address this question, we examined EEG microstate dynamics in the same cohort of preschool children across three consecutive paradigms: Rest, Match, and Oddball. We first identified condition-specific microstate templates and then aligned them across conditions to enable cross-condition comparison within a common labeling framework. Using this approach, we quantified mean duration, time coverage, occurrence, and GEV, and also summarized directed transition probabilities as indices of switching structure. Because templates were extracted within each condition and aligned *post hoc*, condition-related findings were interpreted as descriptive differences in condition-specific microstate profiles rather than as a strict common-template test of condition effects. The analysis addressed three questions: whether preschool microstates show condition-related variation in microstate profiles across the three paradigms; whether conventional temporal microstate features distinguish DLD from TD; and whether group-related differences are more evident in transition organization than in state-wise averages. Given the contrast between the three paradigms, we expected condition-related variation in conventional microstate profiles, more limited group effects in conventional temporal measures, and only exploratory evidence for group-related differences in transition structure.

## 2 Materials and methods

### 2.1 Participants

The final analyzable sample for the present EEG microstate analyses comprised 22 typically developing (TD) children (mean age = 5.00 ± 1.41 years, range = 4.0–7.0 years) and 12 children with developmental language disorder (DLD; mean age = 4.68 ± 0.76 years, range = 4.0–7.0 years). The two groups did not differ significantly in age (*t* = 0.87, *p* = 0.39). These numbers refer to participants who met the EEG quality-control criteria, continuous-segment requirements, and microstate-analysis inclusion criteria for the current study.

All participants were right-handed native Mandarin speakers with normal or corrected-to-normal vision and no reported history of neurological disease, hearing impairment, or autism spectrum disorder. Written informed consent was obtained from the legal guardians of all participants, and the study was approved by the Ethics Committee of Beijing Children’s Hospital, Capital Medical University, National Center for Children’s Health.

DLD was defined as persistent difficulties in language acquisition and use that could not be explained by hearing impairment, intellectual disability, acquired neurological injury, autism spectrum disorder, insufficient language exposure, or other biomedical or environmental factors, consistent with the CATALISE framework (Bishop et al., 2017). Children whose caregivers reported language delay, expressive language difficulties, or broader communication concerns received a standardized DLD assessment. For children aged ≥ 6 years, cognitive ability was assessed using the Wechsler Intelligence Scale for Children—Fourth Edition (WISC-IV), together with standardized Mandarin language assessments based on local norms. Children were included in the DLD group if they had an IQ > 70 and showed a persistent language delay of at least one year in grammar, comprehension, expression, and/or communicative functioning. For children aged < 6 years, the Chinese Neuropsychological and Behavior Scale (CNBS) was administered; children were considered eligible for DLD if they had non-verbal DQ > 70 and verbal DQ ≤ 70, or if standardized Mandarin language testing indicated marked language delay relative to age expectations. The final diagnosis was determined jointly by an experienced developmental-behavioral pediatrician and a research psychologist based on developmental history, caregiver interview, clinical observation, standardized cognitive/developmental assessment, language testing, and DSM-5-informed clinical judgment for language disorder.

TD children were recruited from routine health examinations; their caregivers reported no concerns about language development or communication, and cognitive or developmental performance was age-appropriate (IQ/DQ > 70). Available clinical characteristics of the DLD group, including language difficulty, articulation-related difficulties, stuttering or speech-fluency difficulties, social-communication-related concerns, attention-related concerns, and speech-language therapy status, where available, are summarized in Supplementary Table 3. These associated features were treated as reported clinical characteristics rather than formal comorbid diagnoses unless clinically confirmed. Because the participants were preschool children, and the clinical records contained sensitive information, individual-level raw language scores and detailed clinical records are not publicly reported. Variables that were not systematically documented, such as detailed therapy status or formal severity subtyping, are reported as unavailable where applicable.

The participant cohort and EEG recordings used in the present study were drawn from the same underlying dataset reported previously by Liang et al. (2026). However, the current study addresses a distinct research question and applies a different analytical framework focused on EEG microstate organization, condition-related microstate profiles, and transition-based descriptors. The sample size reported here therefore reflects the final analyzable sample that satisfied the quality-control and inclusion criteria for the current EEG microstate analyses.

### 2.2 Experimental paradigms

To examine EEG microstate dynamics across different cognitive contexts, three continuous EEG recording conditions were employed: a video-based rest-like condition (Rest), a semantic picture–sound matching task (Match), and a Mandarin tone auditory oddball task (Oddball). These paradigms were identical to those reported in Liang et al. (2026), as the present study was based on the same underlying participant cohort and EEG dataset. However, the research questions and analytical framework differed substantially. Whereas the previous study focused primarily on resting-state and task-evoked EEG signatures, the current study investigated EEG microstate organization, condition-dependent microstate dynamics, and directed transition structures. Accordingly, the sample sizes reported here (TD = 22, DLD = 12) refer to the final analyzable participants who passed EEG quality control procedures and met the inclusion criteria for the present microstate analyses, rather than the sample sizes reported in Liang et al. (2026).

All children completed the three recording conditions in a fixed order: Rest (6 min) → Match (approximately 10 min; 4 blocks) → Oddball (approximately 10 min; 2 larger blocks). This fixed sequence was adopted to maintain a consistent and child-friendly testing procedure for preschool-aged participants and to minimize additional variability introduced by procedural changes. Nevertheless, because task order was not counterbalanced, potential order effects, fatigue effects, or habituation effects should be considered when interpreting the findings.

Because preschool children often have difficulty remaining still during conventional eyes-open or eyes-closed resting-state recordings, the Rest condition employed a low-demand passive video-viewing paradigm to improve compliance and reduce movement-related artifacts. Children were seated comfortably and watched a 6-min emotionally neutral silent cartoon (The Mole) without performing any overt response. Consequently, this condition was treated as a rest-like video-viewing baseline rather than a traditional resting-state recording. For simplicity, this condition is referred to throughout the manuscript as the Rest condition.

The Match paradigm employed a picture–sound semantic matching design to elicit language-related semantic processing while minimizing motor demands. The stimulus set consisted of 144 congruent picture–sound pairs and 36 incongruent pairs, yielding an incongruent probability of 20%. Stimuli were presented in a pseudorandom order. Each trial began with a 500-ms fixation cross, followed by the simultaneous presentation of a picture and an auditory stimulus for 750 ms, and then a 2000-ms blank-screen interval. Children were instructed to passively view and listen to the stimuli without making any button presses or verbal responses. The task comprised 180 trials divided into four blocks. To maintain attention and cooperation, a 15-s silent cartoon segment was presented between blocks.

The Oddball paradigm was designed to assess auditory deviance detection under passive listening conditions. Two Mandarin syllables were used: /yi1/ (“衣”; standard stimulus) and /yi3/ (“椅”; deviant stimulus). Stimuli were presented at a comfortable listening volume, with each stimulus lasting approximately 1,000 ms. The task began with an initial mini-block consisting of 20 standard stimuli, followed by eight mixed mini-blocks. Each mixed mini-block contained 20 trials, including 17 standard stimuli and 3 deviant stimuli, resulting in a deviant probability of 15%. Silent cartoon segments lasting 12 s were presented between mini-blocks to help sustain attention. The full set of 180 trials was organized into two larger blocks, separated by a 24-s silent cartoon break. Throughout the Oddball task, children were instructed only to listen passively and were not required to make any overt responses.

Importantly, the EEG data collected during the task conditions were not averaged into event-related potentials (ERPs), nor were ERP components used as the primary outcome measures. Instead, EEG recordings from the Rest, Match, and Oddball conditions were analyzed as continuous EEG time series for microstate analysis. This approach is consistent with the theoretical framework of EEG microstates, which conceptualizes microstates as brief periods of quasi-stable whole-scalp topographies whose temporal expression and transition dynamics are estimated from continuous EEG activity. For the Match and Oddball conditions, task-related continuous EEG segments were retained for microstate feature extraction, whereas silent cartoon intervals and inter-block rest periods were excluded from the analyses.

### 2.3 EEG acquisition and preprocessing

#### 2.3.1 EEG acquisition

EEG was recorded using a 64-channel EGI HydroCel Geodesic Sensor Net (GSN; Electrical Geodesics Inc., Eugene, OR, USA) at a sampling rate of 1,000 Hz, with Cz as the online reference. Electrode impedances were maintained below 50 kΩ throughout the experiment. The dense-array montage was based on the extended international 10–20 system and provided coverage of frontal, central, parietal, occipital, and temporal regions. Children were seated in an adjustable chair facing a computer monitor in a quiet room with soft lighting and were repeatedly reminded to remain still to reduce motion-related artifacts.

#### 2.3.2 EEG preprocessing

All preprocessing was performed in Python using MNE-Python (v1.9; Brunet et al., 2011; Gramfort et al., 2013). The preprocessing pipeline was designed to remove physiological and non-physiological artifacts while preserving the spatiotemporal dynamics relevant for subsequent microstate analysis.

Data import and channel selection. Raw EEG data were imported from EGI .mff files. Non-EEG system, event, and auxiliary channels (e.g., VREF, SESS, CELL, bgin, movi, and STI 014) that were not part of the scalp EEG montage were removed so that subsequent analyses were restricted to EEG channels.

Condition-wise trimming and standardization of usable data. To improve comparability across participants and conditions and to minimize movement-related contamination commonly observed at the beginning and end of recordings in preschool children, continuous EEG was trimmed within each condition before microstate feature extraction. For the Rest condition, a 300-s segment was retained from the middle portion of the recording, excluding the initial settling period and the final segment where movement artifacts tended to increase. For the Match and Oddball conditions, no fixed 300-s window was imposed because the effective task duration was determined by the experimental structure and event markers. Instead, continuous EEG was restricted to active task periods, and non-task intervals, such as silent-animation breaks between blocks or mini-blocks, were excluded whenever applicable.

The retained data duration for each participant and condition was documented after preprocessing. Participants contributed to inferential analyses only when the required condition data were available for the corresponding model; valid sample sizes are reported alongside the results. This procedure was intended to avoid retaining low-quality recordings solely for sample-size balance, which is particularly important in preschool EEG data, where movement-related artifacts are frequent. Because the general preprocessing framework has been described previously (Liang et al., 2026), the present “2 Materials and methods” section focuses on the steps specific to the current study, including condition-specific trimming, condition-specific template extraction, cross-condition alignment, and transition-based analyses.

Resampling and filtering. Data were downsampled from 1,000 Hz to 250 Hz using an anti-aliasing procedure. Line noise was attenuated using a 50-Hz notch filter, followed by a 0.5–60-Hz band-pass filter to remove slow drifts and high-frequency noise.

Bad-channel handling, re-referencing, and ICA-based artifact correction. Bad channels were identified using a combined strategy: (i) acquisition-stage impedance and monitoring notes; (ii) visual inspection for flat channels, excessive noise, or abnormal neighbor relationships; and (iii) automated detection using a RANSAC-based procedure, following principles similar to those implemented in standardized preprocessing frameworks such as PREP (Bigdely-Shamlo et al., 2015). Data were then re-referenced to the average reference.

Artifact correction was performed using extended Infomax independent component analysis (ICA; 20 components; Lee et al., 1999). Because ICA fitting can be affected by low-frequency drift, ICA was fitted on a copy of the data high-pass filtered at 1 Hz, and the resulting unmixing matrix was then applied to the analysis-filtered data (0.5–60 Hz), following established recommendations for ICA-based artifact correction (Winkler et al., 2015). Components were labeled using ICLabel (Pion-Tonachini et al., 2019), and were further reviewed before rejection. Components clearly corresponding to non-neural sources, including ocular, muscle, cardiac, line-noise, or channel-noise components, were removed, whereas components without clear artifactual characteristics were retained. After ICA correction, previously marked bad channels were interpolated using spherical spline interpolation (Perrin et al., 1989).

Channel-set standardization and export. To reduce contamination from non-scalp sensors, neck and face electrodes (E61–E64) were removed after interpolation. EGI channel names were then remapped to standard 10–20 labels, resulting in a standardized 60-channel set. The preprocessed EEG data were exported in BrainVision format for subsequent microstate analysis.

### 2.4 Microstate extraction, cross-condition alignment, and feature computation

**FIGURE 1:**
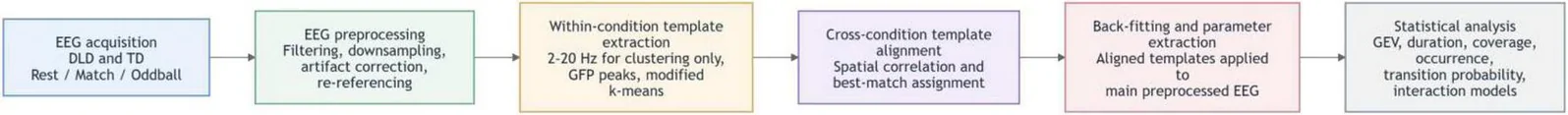
Analytical workflow of EEG microstate analysis across cognitive conditions. Overview of the analytical pipeline used in the present study. Continuous EEG data from Rest, Match, and Oddball were preprocessed and trimmed to artifact-minimized condition-specific segments. For each condition, group-level microstate templates were extracted from GFP peaks using modified k-means clustering. Condition-specific template sets were then aligned across Rest, Match, and Oddball to construct a unified six-topography repertoire (A–F). The aligned templates were back-fitted to each participant’s continuous EEG to derive conventional microstate features, including mean duration, time coverage, occurrence rate, and global explained variance (GEV). Directed transition probability matrices and cross-condition Δ-transition indices were then computed as higher-order descriptors of switching organization, followed by primary, descriptive, and exploratory statistical analyses.

The overall analytical workflow is illustrated in Figure 1. EEG microstate analysis was performed using Cartool (Denis Brunet, University of Geneva; v1.9) and interpreted in line with current methodological recommendations for temporo-spatial EEG analysis and contemporary microstate research (Murray et al., 2008; Michel et al., 2024; Kalburgi et al., 2024). Microstates were defined as recurring quasi-stable scalp potential topographies that summarize continuous EEG as a sequence of rapidly alternating large-scale brain states (Pascual-Marqui et al., 1995; Khanna et al., 2015; Michel et al., 2024). The analysis comprised four steps: within-condition template extraction, cross-condition alignment, back-fitting, and feature quantification. This condition-specific framework was used to describe the dominant topographic repertoire within each cognitive context, rather than to provide a conservative common-template test of condition effects.

#### 2.4.1 Within-condition template extraction and selection of the number of classes

To enhance the dominant topographic patterns relevant for microstate extraction, an additional 2–20 Hz band-pass filter was applied to each condition (Rest, Match, and Oddball) for clustering only; back-fitting and feature computation were performed on the main preprocessed data (0.5–60 Hz; Section “2.3 EEG acquisition and preprocessing”). Global field power (GFP) was computed, and only scalp maps at local GFP peaks were entered into clustering to improve the signal-to-noise ratio and reduce temporal redundancy. Within each condition, GFP-peak topographies were clustered using Cartool’s modified k-means algorithm with polarity ignored.

To justify the retained number of classes, candidate solutions with neighboring values of k were evaluated separately for Rest, Match, and Oddball. Model selection was based on Cartool’s Meta-Criterion, global explained variance (GEV), model parsimony, and the feasibility of subsequent cross-condition topographic alignment. Because the primary objective was to compare microstate parameters and transition matrices across conditions, a common within-condition number of classes was used for the primary analyses.

In the present dataset, the preferred k value was not identical across conditions. *k* = 5 provided a consistent improvement over *k* = 4 and reduced the risk of underfitting, whereas higher-k solutions increased the risk of over-segmentation, condition-specific fragmentation, and unstable cross-condition label matching. Therefore, *k* = 5 was retained as a cross-condition compromise for the primary analyses rather than as the absolute optimal solution for every condition. GEV and Meta-Criterion values for neighboring-k solutions are reported in Supplementary Table 1.

#### 2.4.2 Cross-condition alignment and construction of a unified six-topography repertoire

To enable interpretable cross-condition comparisons under a unified labeling framework, the condition-specific five-template solutions were aligned at the group-template level. Spatial similarity between templates from different conditions was quantified using polarity-invariant Pearson spatial correlation. Cross-condition correspondences were established using a one-to-one maximum-correlation assignment to avoid many-to-one label collapse and reduce ambiguity. This procedure yielded a unified six-topography repertoire (A–F), comprising condition-shared microstates (C–E) and condition-specific microstates (A, B, and F) that did not consistently emerge across all conditions. Because some condition-specific templates showed clear cross-condition correspondence, whereas others remained condition-restricted, the final aligned repertoire contained six templates rather than five. This alignment strategy was intended to improve cross-condition interpretability while retaining the empirical structure of the condition-specific solutions (Koenig et al., 2024).

It should be noted that this condition-specific template strategy was intended to characterize the dominant topographic organization within each cognitive context. It was not designed to provide the most conservative test of condition effects within a single common template space. Because templates were extracted separately within each condition and then aligned *post hoc*, between-condition differences in conventional microstate parameters, especially those involving condition-dominant topographies such as A, B, and F, should be interpreted descriptively rather than as definitive evidence for independent task-specific microstate classes. A pooled group-level or meta-microstate extraction would provide a more conservative framework for testing condition-related modulation within a shared template space.

#### 2.4.3 Back-fitting and conventional feature computation

The aligned templates (A–F) were back-fitted to each participant’s continuous EEG separately within each condition. At each time point, the scalp topography was correlated with each template using polarity-invariant Pearson correlation, and the time point was assigned to the template with the highest correlation, resulting in a condition-specific microstate label time series for each participant. For each microstate and condition, the following conventional features were computed at the participant level: (i) global explained variance (GEV), (ii) mean duration (ms), (iii) time coverage (%) and (iv) occurrence rate (events/s; segment density). These measures capture complementary aspects of microstate stability, occupancy, recruitment, and explained variance (Khanna et al., 2015; Michel et al., 2024; Tarailis et al., 2024).

#### 2.4.4 Transition matrices and Δ-transition indices

To characterize higher-order switching organization, we computed directed microstate transition probability matrices from the back-fitted label time series, excluding self-transitions (i → i). For each participant and condition, transition probabilities were row-normalized by the total number of outgoing transitions from each source state. Because each condition expressed a partially overlapping subset of the aligned six-topography repertoire, full transition matrices were summarized descriptively within condition, whereas cross-condition Δ-transition analyses were restricted to transitions between microstates shared by the two contrasted conditions.

To quantify condition-related changes in switching organization more directly, we additionally computed within-child Δ-transition indices for each shared transition probability across the three condition pairs: Match–Rest (MR), Oddball–Rest (OR), and Oddball–Match (OM). Specifically, MR contrasts were evaluated within the B/C/D/E subspace, OR contrasts within the A/C/D/E subspace, and OM contrasts within the C/D/E/F subspace. Because each contrast involved four shared microstates, each Δ-transition contrast included 12 directed non-self-transition pathways. Across the three contrasts, this yielded 36 Δ-transition pathways in total, comprising three contrast-specific sets of 12 pathways each.

This distinction between conventional state-wise measures and higher-order transition-based descriptors was motivated by previous work suggesting that sequence-level complexity and switching organization may provide additional information beyond traditional temporal microstate summaries (Stevens and Kircher, 1998; Tait et al., 2020; Wu et al., 2025). Accordingly, Δ-transition indices were used as exploratory descriptors of condition-related changes in microstate switching organization rather than as confirmatory markers of DLD-related abnormalities.

### 2.5 Conventional microstate features and transition-based descriptors

The analyses were organized hierarchically. First, conventional microstate features, including mean duration, time coverage, occurrence rate, and global explained variance (GEV), were treated as the predefined primary outcome measures. These indices were used to characterize the temporal expression and explanatory contribution of each microstate class in the continuous EEG recordings. Because microstate templates were extracted within each condition and then aligned across conditions, condition-related effects were interpreted descriptively as task-context-related differences in condition-specific microstate profiles rather than as a strict common-template test of condition effects.

Second, full directed transition probability matrices were examined as complementary descriptive measures of microstate switching organization. Third, Δ-transition indices were calculated as exploratory follow-up descriptors of condition-related changes in directed transition probabilities. Because transition matrices contain a relatively large number of directed pathways and microstate syntax analysis remains methodologically developing, transition-level analyses were treated as exploratory and hypothesis-generating rather than confirmatory (Haydock et al., 2025). Omnibus ANOVA results for conventional microstate features are summarized in Table 1, whereas representative exploratory Δ-transition pathways are summarized in Table 2.

**TABLE 1:** Mixed-design repeated-measures ANOVA results for conventional microstate parameters across groups and conditions.

Feature	State	Effect	F (df1, df2)	p_final	np2
MeanDur	C	Group	0.02 (1, 32)	0.890	0.001
MeanDur	C	Condition	34.33 (2, 64)	< 0.001	0.518
MeanDur	C	Interaction	1.34 (2, 64)	0.268	0.040
MeanDur	D	Group	0.51 (1, 32)	0.482	0.016
MeanDur	D	Condition	1.31 (2, 64)	0.277	0.039
MeanDur	D	Interaction	1.38 (2, 64)	0.259	0.041
MeanDur	E	Group	0.23 (1, 32)	0.574	0.010
MeanDur	E	Condition	63.44 (2, 64)	< 0.001	0.665
MeanDur	E	Interaction	0.91 (2, 64)	0.406	0.028
TimeCov	C	Group	0.27 (1, 32)	0.605	0.008
TimeCov	C	Condition	69.86 (2, 64)	< 0.001	0.686
TimeCov	C	Interaction	0.29 (2, 64)	0.750	0.009
TimeCov	D	Group	3.57 (1, 32)	0.068	0.100
TimeCov	D	Condition	2.07 (2, 64)	0.149	0.061
TimeCov	D	Interaction	0.66 (2, 64)	0.523	0.020
TimeCov	E	Group	0.04 (1, 32)	0.839	0.001
TimeCov	E	Condition	82.72 (2, 64)	< 0.001	0.721
TimeCov	E	Interaction	0.12 (2, 64)	0.898	0.003
SegDensity	C	Group	0.01 (1, 32)	0.929	0.000
SegDensity	C	Condition	37.50 (2, 64)	< 0.001	0.540
SegDensity	C	Interaction	0.20 (2, 64)	0.816	0.006
SegDensity	D	Group	4.86 (1, 32)	0.035	0.132
SegDensity	D	Condition	2.09 (2, 64)	0.144	0.061
SegDensity	D	Interaction	0.54 (2, 64)	0.587	0.017
SegDensity	E	Group	0.00 (1, 32)	0.972	0.000
SegDensity	E	Condition	66.86 (2, 64)	< 0.001	0.676
SegDensity	E	Interaction	0.05 (2, 64)	0.948	0.002
Gev	C	Group	1.72 (1, 32)	0.199	0.051
Gev	C	Condition	9.71 (2, 64)	< 0.001	0.233
Gev	C	Interaction	0.25 (2, 64)	0.778	0.008
Gev	D	Group	0.33 (1, 32)	0.568	0.010
Gev	D	Condition	21.67 (2, 64)	< 0.001	0.404
Gev	D	Interaction	1.12 (2, 64)	0.332	0.034
Gev	E	Group	0.06 (1, 32)	0.811	0.002
Gev	E	Condition	0.15 (2, 64)	0.865	0.005
Gev	E	Interaction	0.62 (2, 64)	0.539	0.019

Analyses were conducted on the final analyzable sample of 22 TD children and 12 children with DLD. Feature indicates the conventional microstate parameter tested, and State indicates the aligned microstate label. Effect refers to the omnibus ANOVA effect tested: Group, Condition, or Group × Condition. Group was the between-subject factor (DLD vs. TD), and Condition was the within-subject factor (Rest, Match, Oddball). F (df1, df2) reports the F statistic with corresponding degrees of freedom. p_final indicates the *p*-value used for statistical interpretation; for effects involving Condition, Greenhouse–Geisser correction was applied when the sphericity assumption was violated. ηp^2^, partial eta squared.

**TABLE 2:** Exploratory group comparisons of representative candidate Δ-transition pathways across condition contrasts.

Contrast	Transition	TD	DLD	Mean diff	*t*	*p*	q	g
MR	C→B	−0.094 ± 0.049	−0.126 ± 0.075	−0.033	−1.363	0.191	0.984	−0.538
MR	E→B	−0.063 ± 0.073	−0.088 ± 0.114	−0.026	−0.700	0.494	0.984	−0.279
OM	E→D	0.003 ± 0.020	0.036 ± 0.045	0.033	2.382	0.033	0.393	1.030
OM	D→E	0.060 ± 0.040	0.077 ± 0.051	0.018	1.029	0.317	0.877	0.388
OR	E→A	−0.109 ± 0.048	−0.088 ± 0.059	0.021	1.073	0.297	0.900	0.401
OR	A→C	−0.236 ± 0.060	−0.208 ± 0.077	0.027	1.066	0.300	0.900	0.403

Values for TD and DLD are presented as mean ± SD of Δ-transition indices. Mean diff indicates DLD - TD; therefore, positive values indicate higher Δ-transition values in the DLD group, whereas negative values indicate higher Δ-transition values in the TD group. Welch’s *t* statistics, uncorrected *p*-values, Benjamini–Hochberg false discovery rate-corrected q values, and Hedges’ g effect sizes are reported for each representative candidate pathway. The six pathways shown here were selected for compact exploratory reporting from the full Δ-transition analysis and do not define the correction family. FDR correction was applied separately within each contrast-specific family of 12 pathways. No displayed pathway survived FDR correction. MR, Match–Rest; OR, Oddball–Rest; OM, Oddball–Match.

#### 2.5.1 Statistical analysis

All statistical analyses were conducted in IBM SPSS Statistics 27 and Python-based scripts. Statistical significance was set at two-tailed *p* < 0.05, with correction for multiple comparisons applied within predefined analysis families.

Primary analyses focused on conventional microstate features (GEV, mean duration, time coverage, and occurrence rate) as the predefined outcome measures. These features were analyzed using a mixed-design repeated-measures ANOVA with Group (DLD vs. TD) as the between-subject factor and Condition (Rest, Match, Oddball) as the within-subject factor. Sphericity was assessed using Mauchly’s test; when violated, Greenhouse–Geisser correction was applied, and corrected degrees of freedom are reported. Effect sizes are reported as partial eta squared (ηp^2^). When significant Condition effects or Group × Condition interactions were observed, Bonferroni-adjusted *post hoc* pairwise comparisons were performed for the Match–Oddball, Match–Rest, and Oddball–Rest contrasts. Group effects and Group × Condition interactions were interpreted as the primary tests of DLD–TD differences within this analytical framework.

For transition-based analyses, directed transition probability matrices were computed for each participant and condition, excluding self-transitions. Full transition matrices were summarized descriptively at the group level to characterize switching architecture across Rest, Match, and Oddball and to visualize potential group-related deviations. These matrices were not treated as primary confirmatory inferential outcomes.

Δ-transition indices were calculated for three condition contrasts: Match–Rest (MR), Oddball–Rest (OR), and Oddball–Match (OM). Because each contrast was restricted to the four microstates shared by the two contrasted conditions, each contrast included 12 directed non-self-transition pathways, yielding 36 Δ-transition pathways across the full testing family. Participant-level Δ-transition values were compared between the DLD and TD groups using Welch’s independent-samples *t*-tests, and effect sizes were quantified using Hedges’ g. Benjamini–Hochberg false discovery rate correction was applied separately within each of the three contrast-specific families of 12 pathways. Therefore, corrected q values reflect correction across all 12 pathways within the corresponding condition contrast rather than only the representative pathways reported in Table 2.

Because reporting all transition pathways in the main text would be unwieldy, Table 2 presents six representative candidate Δ-transition pathways for concise reporting. Specifically, two pathways were selected from each condition to illustrate the most prominent and interpretable descriptive group-divergence patterns observed in the full DLD–TD Δ-transition difference matrices. This selection was used only for compact presentation and interpretation; it did not define the multiple-comparison correction family and did not constitute a separate confirmatory testing procedure. Accordingly, any pathway showing nominal uncorrected significance but failing to survive FDR correction was interpreted as exploratory and hypothesis-generating rather than as robust evidence of a group difference.

Given the modest final analyzable sample size, particularly in the DLD group, group-level effects were interpreted with caution. The present study was not designed or powered to provide a definitive test of small-to-moderate DLD–TD differences. As an approximate between-group sensitivity reference, the final sample size of 12 children with DLD and 22 TD children would provide 80% power at α = 0.05 only for relatively large between-group effects, approximately Cohen’s d ≈ 1.0. Therefore, non-significant corrected group effects were not interpreted as evidence for the absence of group-related neural differences. Instead, group-level findings were evaluated by considering both statistical evidence and effect-size estimates. Nominal or near-threshold effects were treated as suggestive rather than confirmatory unless they survived the relevant correction procedure (Button et al., 2013; Larson and Carbine, 2017; Baker et al., 2021).

All primary statistical analyses were conducted using the final analyzable sample for the present EEG microstate study, comprising 22 TD children and 12 children with DLD. This sample reflects participants who met the EEG quality-control, continuous-segment selection, and microstate-analysis inclusion criteria for the current study. For each inferential model, participants were included only when the required condition data were available after preprocessing and trimming. Valid sample sizes were reported alongside the corresponding analyses where applicable.

## 3 Results

### 3.1 Cross-condition alignment of condition-specific microstate templates

**FIGURE 2:**
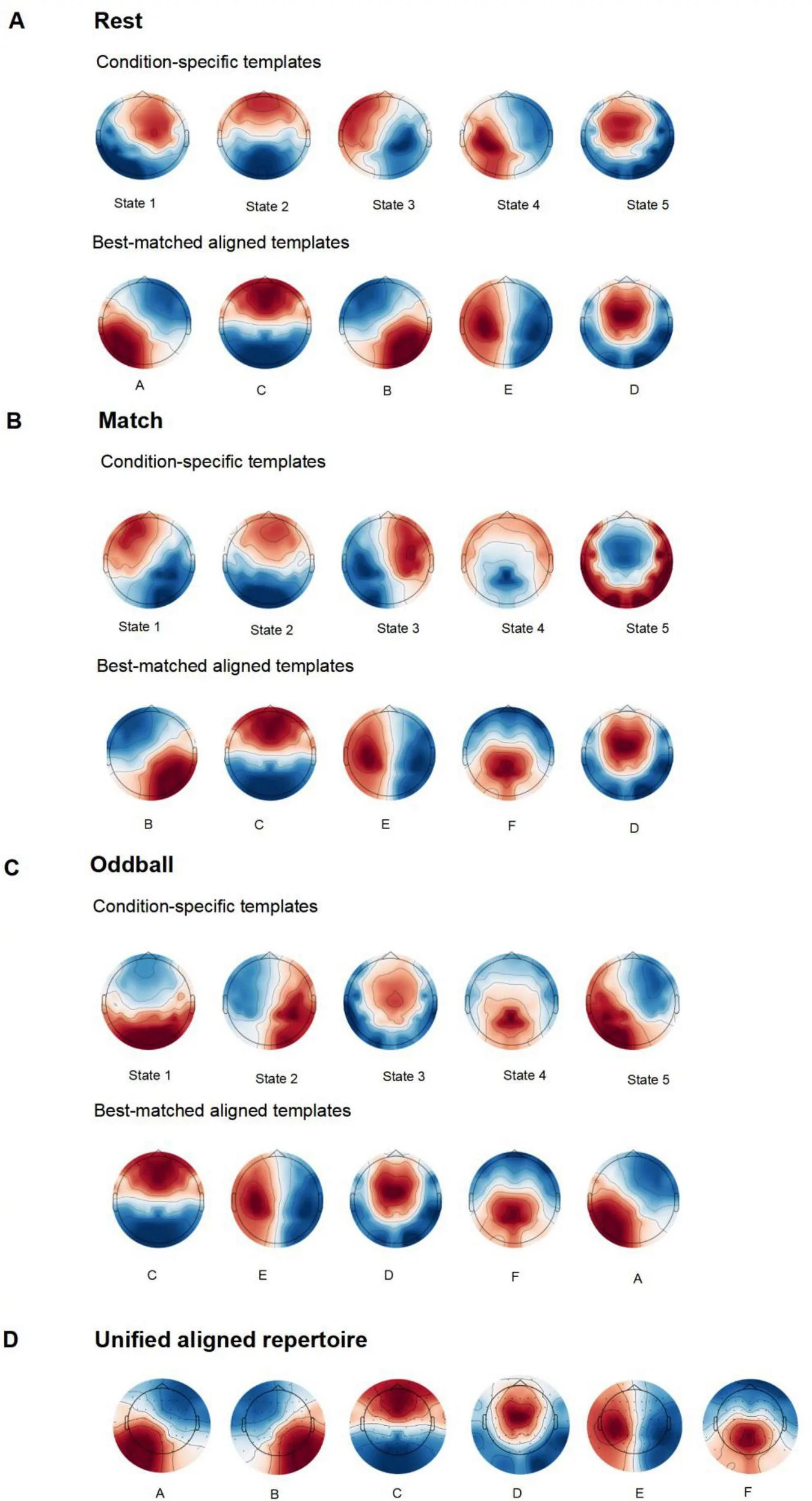
Cross-condition alignment of condition-specific microstate templates and construction of a unified template repertoire. For each condition, five condition-specific microstate templates were identified from GFP-peak topographies using modified k-means clustering. Panels **(A–C)** show the original condition-specific templates for Rest, Match, and Oddball, respectively (top row in each panel), together with their best-matched aligned templates after cross-condition topographic matching (bottom row in each panel). Template matching was based on spatial similarity of scalp topographies with polarity ignored. The aligned labels indicate the closest correspondence between condition-specific templates and the common template set. Panel **(D)** shows the final unified aligned repertoire comprising six templates (A–F), which was derived from the cross-condition alignment procedure and used for subsequent comparative analyses.

Condition-specific clustering yielded stable five-template solutions for Rest, Match, and Oddball. Visual inspection and spatial matching indicated that several scalp topographies were shared across conditions, whereas others were condition-specific or only partially overlapping in configuration. To support cross-condition interpretation within a unified aligned framework, the condition-specific template sets were therefore aligned according to their best topographic correspondence, yielding a unified six-topography repertoire (A–F) that served as the reference framework for downstream analyses (Figure 2). This aligned repertoire comprised condition-shared microstates (C–E) and condition-specific or condition-dominant microstates (A, B, and F), consistent with the partial overlap of the original condition-specific clustering solutions. Because templates were extracted within each condition and then aligned *post hoc*, these topographic findings were interpreted as descriptive evidence of task-context-related organization rather than as a conservative common-template test of condition effects. This aligned repertoire served as the unified reference framework for all subsequent cross-condition analyses.

### 3.2 Condition-related differences in condition-specific microstate temporal profiles

We next examined whether conventional microstate features varied across the three recording conditions. Mixed-design repeated-measures ANOVA revealed significant main effects of Condition across multiple conventional microstate parameters, whereas the main effect of Group and the Group × Condition interaction were generally small and non-significant (Table 1). Across the reported measures, one aligned state showed stronger expression during Match, whereas other shared states were more prominent during Rest and Oddball. This overall pattern was consistent across mean duration, time coverage, occurrence rate, and GEV, indicating that preschool microstate profiles varied across task contexts under the present condition-specific template framework.

**FIGURE 3:**
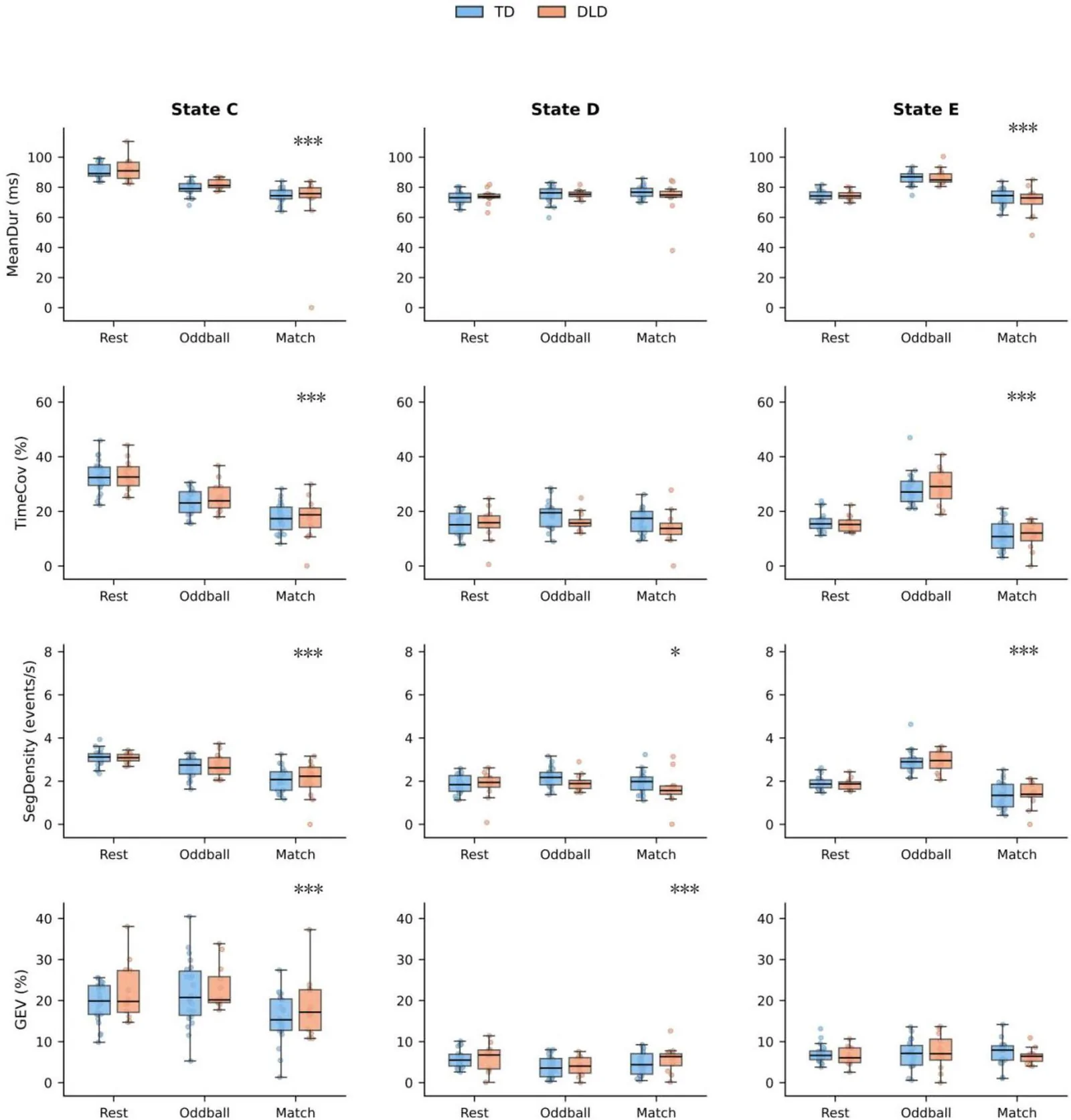
Microstate temporal parameters across conditions and groups for aligned microstates. Participant-level distributions of mean duration (MeanDur), time coverage (TimeCov), segment density (SegDensity), and global explained variance (GEV) are shown for the aligned microstates C–E across Rest, Oddball, and Match. Boxplots summarize TD and DLD distributions, with individual participant values overlaid. Boxes indicate the interquartile range with the median, and whiskers represent 1.5 × IQR. Panel-level significance markers denote omnibus Condition effects (*p < 0.05, ***p < 0.001). Final significance marker placement follows the corrected omnibus Condition *p*-values reported in Table 1.

Mean duration suggested that the temporal stability of several states differed across paradigms. Time coverage and occurrence rate further showed stronger recruitment of the Match-dominant state during Match, whereas other shared states contributed more during Rest and Oddball. GEV showed a similar pattern, indicating that the contribution of individual microstates to the global field structure also changed across conditions. Across the reported measures, the results indicate condition-related differences in condition-specific microstate temporal profiles across Rest, Match, and Oddball (Table 1 and Figure 3). Because the primary analysis was based on within-condition template extraction followed by cross-condition alignment, these findings should be interpreted as descriptive evidence of task-context-related topographic organization rather than as definitive evidence for independent task-specific microstate classes or as a strict common-template test of condition effects.

### 3.3 Limited evidence for DLD–TD differences in temporal parameters

In contrast to the condition-related differences described above, evidence for between-group differences in conventional temporal microstate features was limited. Across the reported parameters, neither the main effect of Group nor the Group × Condition interaction provided robust statistical evidence for group-related differences (Table 1). These results indicate that, within the present sample and analytical framework, the overall pattern of condition-specific microstate profiles was broadly similar in the DLD and TD groups. This finding should not be interpreted as evidence that neural dynamics are unaffected in DLD. Rather, it indicates that the present study did not identify robust DLD–TD differences at the level of conventional microstate parameters. Given the modest sample size and the heterogeneity of pediatric EEG data, small-to-moderate group effects cannot be excluded.

### 3.4 Descriptive transition probability matrices

**FIGURE 4:**
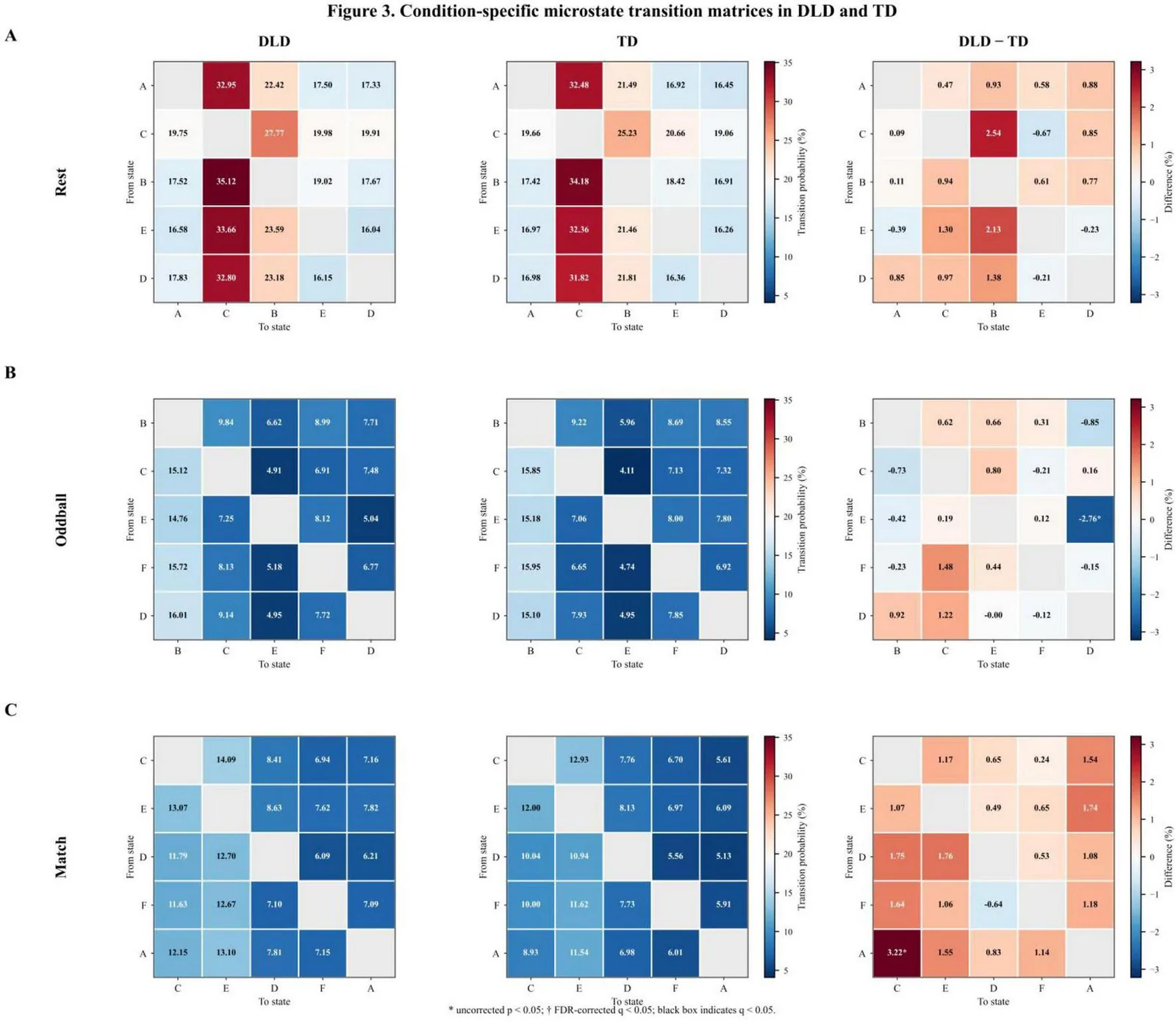
Transition probability matrices across conditions and group-difference patterns. Directed microstate transition probability matrices for **(A)** Rest, **(B)** Oddball, and **(C)** Match, shown separately for the TD and DLD groups, together with corresponding group-difference matrices *(*DLD - TD*)*. Rows indicate source states, and columns indicate target states; self-transitions *(*i → i*)* were excluded. Transition probabilities were row-normalized within each source state. The overall transition architecture differed across cognitive conditions, indicating condition-dependent reorganization of switching structure. Between-group deviations were generally small in magnitude, and candidate pathway analyses identified only limited exploratory evidence for pathway-specific group differences. Asterisks indicate uncorrected *p* < 0.05 for candidate pathways selected for exploratory follow-up.

We then examined transition-based descriptors of switching organization using full directed transition probability matrices. At the descriptive level, the dominant transition architecture differed across Rest, Match, and Oddball, indicating condition-related variation in switching structure (Figure 4). However, the between-group difference matrices (DLD–TD) suggested that most deviations were small in magnitude, typically within only a few percentage points, and did not indicate an obvious qualitative disruption of the overall transition pattern. The full transition matrices were therefore treated primarily as descriptive summaries of switching organizations. Given the large number of directed pathways and the developing status of microstate syntax analysis, these transition-level results were not treated as primary confirmatory evidence.

### 3.5 Exploratory Δ-transition pathway analyses

To quantify condition-related changes in switching organization more directly, we next examined candidate Δ-transition pathways derived from the Match-Rest, Oddball-Rest, and Oddball-Match contrasts within the relevant shared-state subspaces. Specifically, MR contrasts were evaluated within the B/C/D/E subspace, OR contrasts within the A/C/D/E subspace, and OM contrasts within the C/D/E/F subspace. Because each contrast involved four shared microstates, each contrast included 12 directed non-self-transition pathways, yielding 36 Δ-transition pathways across the full testing family. All pathways were included in the Benjamini–Hochberg FDR correction. See Supplementary Table 2 for details.

Table 2 reports six representative candidate Δ-transition pathways to provide a compact summary of the most prominent and interpretable descriptive DLD–TD divergence patterns across the MR, OR, and OM contrasts. Importantly, these six pathways were selected for presentation only. They were not the only pathways tested, and they did not define the multiple-comparison correction family. The reported q values therefore reflect FDR correction within the corresponding 12-path contrast-specific family rather than correction across only the six pathways shown in Table 2.

These follow-up analyses provided only limited evidence for pathway-specific group differences. In the Oddball-Match contrast, the E→D transition showed an uncorrected between-group difference, with a higher Δ-transition value in the DLD group than in the TD group. Specifically, the OM E→D pathway showed a nominal uncorrected group difference (TD: 0.003 ± 0.020; DLD: 0.036 ± 0.045; mean difference = 0.033; *p* = 0.033; Hedges’ g = 1.030), but this effect did not survive FDR correction within its corresponding contrast-specific family (*q* = 0.393). No other candidate pathway reached the uncorrected significance threshold across the remaining contrasts. The transition-based analyses indicated condition-related variation in switching organization at the descriptive level, but no FDR-corrected evidence for reliable DLD–TD differences in pathway-specific switching organization. Accordingly, these findings should be interpreted as exploratory and hypothesis-generating rather than as confirmatory evidence for DLD-related abnormalities in transition organization.

### 3.6 Summary of results

Taken together, the most consistent observation was that condition-specific microstate profiles differed across Rest, Match, and Oddball. These differences suggest that preschool EEG microstate organization is sensitive to task context, but because templates were extracted within each condition and aligned *post hoc*, the condition-related findings should be interpreted descriptively. In contrast, evidence for DLD–TD differences in conventional microstate parameters was limited. Transition matrices and Δ-transition analyses provided complementary exploratory information but did not yield FDR-corrected evidence for reliable group differences.

## 4 Discussion

### 4.1 Principal findings

This study examined whether preschool EEG microstates vary across different cognitive contexts and whether DLD-related differences are more evident in transition structure than in conventional temporal measures. Three results were most evident in the present dataset. Microstate profiles differed across Rest, Match, and Oddball. Group differences in conventional temporal features were limited. Transition analyses also showed task-context-related variation in switching structure at the descriptive level, although no Δ-transition pathway survived correction across the full testing family. The dominant pattern in the present dataset was task-context-related variation in condition-specific microstate profiles rather than robust DLD–TD separation. Because templates were extracted within each condition and aligned *post hoc*, the condition-related findings should be interpreted descriptively rather than as definitive evidence for independent task-specific microstate classes or as a strict common-template test of condition effects.

### 4.2 Task-context-related differences in preschool microstate profiles

The main finding of the present study was that conventional EEG microstate profiles varied across Rest, Match, and Oddball under the condition-specific template extraction and cross-condition alignment framework. Across conventional temporal parameters, the relative expression of several aligned states differed across Rest, Match, and Oddball. This pattern indicates task-context-related differences in the temporal expression of large-scale scalp field topographies. One task-dominant state showed stronger expression during Match, as reflected by higher coverage, occurrence, and GEV, whereas other shared states were expressed more strongly during Rest and Oddball. Mean duration also varied across conditions for most states, indicating that task-related modulation affected not only how often a state was recruited but also how long it remained active once entered. The clearest signal in the present dataset was therefore condition-related variation in microstate profiles rather than a robust disorder effect. Importantly, because templates were extracted separately within each condition and then aligned *post hoc*, these findings should be regarded as descriptive differences in condition-specific microstate profiles rather than as a strict common-template test of condition effects.

These findings are consistent with current views of EEG microstates as transient large-scale scalp field topographies whose temporal expression is shaped by ongoing perceptual, attentional, and cognitive context (Khanna et al., 2015; Michel et al., 2024; Tarailis et al., 2024). Methodological work has also emphasized that microstate features should not be treated as fixed entities with the same functional meaning across contexts. Rather, they are temporospatial summaries whose expression depends on both cognitive state and analytic approach (Kalburgi et al., 2024; Koenig et al., 2024; Michel et al., 2024). The condition-related differences observed here are therefore plausible within this methodological and developmental context. Rest-like viewing, semantic picture–sound matching, and passive auditory oddball listening differ in perceptual and cognitive demands. The associated large-scale EEG topographies would therefore not be expected to show identical temporal organization across conditions.

From a developmental perspective, the present findings add to the limited literature on EEG microstates in early childhood. Although microstate analysis has been applied in infants and young children, most developmental studies have focused on resting-state activity or single-task settings. Much less is known about how preschool children show different microstate profiles across different task contexts. The present results suggest that even in early childhood, microstates are not merely background activity but large-scale spatiotemporal patterns that may vary across rest-like viewing, semantic matching, and auditory deviance detection. This provides a more developmentally grounded and state-sensitive context for interpreting atypical neural dynamics in DLD.

### 4.3 Limited group differences in conventional temporal parameters

In contrast to the condition-related differences described above, group differences in conventional temporal parameters were limited. Neither the main effect of Group nor the Group × Condition interaction provided robust statistical evidence for DLD–TD differences, and condition-wise *post hoc* comparisons did not survive correction for multiple testing. The absence of robust group effects does not necessarily indicate preserved neural dynamics in DLD. Instead, it suggests that the present study did not obtain sufficiently stable evidence for broad group differences in the average temporal expression of individual microstates. Measures such as duration, coverage, occurrence, and GEV summarize each state separately and are therefore most sensitive to relatively large state-wise biases. If DLD-related differences are more distributed, context-dependent, or sequentially organized, they may remain weakly expressed in state-wise summaries. Given the modest final analyzable sample size and the heterogeneity of pediatric EEG data, small-to-moderate DLD-related effects cannot be excluded.

The present findings are broadly consistent with prior EEG findings in DLD and related language-impairment populations. Resting-state and task-based studies have reported atypical neural signatures in children with DLD, including altered speech tracking and differences in baseline oscillatory organization, but such effects are not always expressed as large and stable group differences in a single summary metric (Liang et al., 2026; Nora et al., 2024; Stanojevic et al., 2023). Broader syntheses of DLD-related EEG and intervention studies likewise suggest heterogeneity in both the neural measures used and the form of group-related divergence, which may vary with task demands, developmental stage, and level of analysis (Hu et al., 2025). The limited group differences in conventional microstate features are informative rather than simply null findings. They suggest that the broad temporal expression of aligned microstates may be relatively similar at the group level, while subtler, state-specific, clinically heterogeneous, or transition-level differences remain possible.

### 4.4 Transition organization as a complementary exploratory level

The transition analyses were used as a complementary exploratory level of analysis beyond conventional temporal summaries. At the descriptive level, full transition matrices differed across Rest, Match, and Oddball, again indicating task-context-related variation in the higher-order switching architecture. Between-group deviations, however, were generally small, and only one pathway showed an uncorrected difference in relation to Oddball processing. The results suggest that the large-scale transition structure was broadly similar in DLD and TD children, while the nominal pathway-level difference did not provide corrected evidence for a reliable DLD-related transition abnormality.

The present transition findings are consistent with a growing literature suggesting that clinically relevant information may be carried not only by first-order temporal descriptors but also by higher-order sequence-level properties of microstate dynamics. For example, microstate transition complexity has been reported to aid early Alzheimer’s disease classification (Tait et al., 2020), and more recent work has shown that entropy-related and sequence-sensitive descriptors may complement conventional microstate features in characterizing mild cognitive impairment (Wu et al., 2025). Methodological reviews have likewise noted that microstate syntax and sequence-level organization may provide useful information beyond state-wise averages, while also emphasizing challenges in how such metrics are defined, selected, and interpreted (Haydock et al., 2025). In the present study, the findings are compatible with the possibility that DLD-related atypicality may involve switching organization, but the current evidence remains exploratory and should not be interpreted as showing that transition descriptors are superior to conventional microstate measures.

These findings nevertheless require cautious interpretation. In the current dataset, the evidence was limited and largely exploratory, and the candidate pathway analyses were designed as follow-up tests derived from descriptive DLD–TD difference matrices rather than as the primary confirmatory inferential target. All Δ-transition pathways were included in the full-family FDR correction, and the nominal OM E→D effect did not survive correction. Transition-based descriptors may prove more sensitive in future work, but the present results do not support a strong claim that they are superior to conventional temporal measures in DLD. Instead, they identify transition organizations as a promising level of analysis for further testing in larger pediatric datasets. This view is consistent with recent methodological reviews, which emphasize that pairwise transitions can be informative but require careful inferential framing and replication (Haydock et al., 2025).

### 4.5 Limitations and future directions

Several limitations should be noted. First, the final analyzable sample was modest, particularly in the DLD group. Strict EEG quality control helped ensure that the continuous EEG data were suitable for microstate analysis, but it also reduced the number of participants available for statistical testing. Accordingly, the present study was not powered to provide a definitive test of small-to-moderate DLD–TD differences. Non-significant corrected group effects should therefore not be interpreted as evidence that neural dynamics are unaffected in children with DLD. Rather, they indicate that the present study did not obtain sufficiently stable evidence for clear group differences under the current sample size, data-quality criteria, and multiple-comparison framework (Button et al., 2013; Larson and Carbine, 2017). Future studies should include *a priori* power planning and should report EEG retention rates, usable data duration, and analyzable segment counts after quality control, because statistical power in pediatric EEG research depends not only on participant number but also on the amount and quality of usable EEG data (Baker et al., 2021).

Second, the present DLD sample was clinically heterogeneous. Although all children with DLD met the study’s diagnostic criteria, had nonverbal IQ/DQ scores above 70, and had no reported history of hearing impairment, clear neurological disease, autism spectrum disorder, or marked intellectual/global developmental delay, available clinical records indicated variability in language and associated clinical features. Some children had articulation-related difficulties, stuttering or speech-fluency difficulties, social-communication-related concerns, or attention-related concerns. These characteristics should be interpreted as associated clinical features or reported concerns rather than formal comorbid diagnoses unless clinically confirmed. Because of the modest sample size, we could not examine whether expressive versus receptive–expressive profiles, language severity, articulation problems, fluency difficulties, attention-related features, or treatment status were associated with distinct microstate patterns. Future DLD microstate studies should therefore characterize language phenotype, severity, associated clinical features, and speech-language therapy status more systematically.

Third, the resting condition was implemented as a passive viewing, rest-like state to improve compliance in preschool children. Although developmentally appropriate, this approach differs from canonical eyes-open or eyes-closed resting-state paradigms and may influence the observed temporal and topographic structure. Thus, Rest in the present study should be understood as a low-demand video-viewing baseline rather than a canonical resting-state recording.

Fourth, because condition-specific clustering solutions were not fully identical, cross-condition comparison required alignment to a unified repertoire, introducing an additional interpretive step even though it provided a principled framework for comparison. Because templates were extracted separately for Rest, Match, and Oddball, some between-condition differences in conventional microstate parameters may partly reflect the condition-specific extraction and alignment procedure, especially for condition-dominant topographies such as A, B, and F. Therefore, the condition-related findings should be interpreted as descriptive evidence of task-context-related microstate organization rather than as definitive evidence for independent task-specific microstate classes or as a strict common-template test of condition effects. Future studies should use pooled group-level or meta-microstate approaches to determine whether these condition-related patterns persist when all conditions are evaluated within a shared template space.

Finally, transition-based analyses should be interpreted cautiously. The Δ-transition analyses were exploratory and hypothesis-generating, and no pathway survived correction within its corresponding contrast-specific family. Thus, the nominal OM E→D finding should not be considered robust evidence of a DLD-related transition abnormality. Future work should examine predefined transition metrics, microstate syntax, entropy, non-randomness, and other complexity-based indices in larger and independent samples, with appropriate correction for comparison families (Haydock et al., 2025; Tait et al., 2020; Wu et al., 2025). More broadly, integrating microstate analysis with EEG markers already implicated in DLD, such as speech tracking, mismatch responses, and resting-state oscillatory signatures, may help build a more complete developmental framework of neural dynamics in language disorder (Liang et al., 2026; Nora et al., 2024; Stanojevic et al., 2023). The present study should therefore be viewed as a state-sensitive descriptive account showing that preschool EEG microstate profiles vary across cognitive contexts, while evidence for DLD-specific microstate alterations remains limited and requires confirmation in larger, better-characterized samples.

## 5 Conclusion

The present study examined that EEG microstate dynamics in preschool children vary across Rest, Match, and Oddball, suggesting task-context-related differences in condition-specific microstate profiles. Because the present analysis used condition-specific template extraction followed by cross-condition alignment, these condition-related findings should be interpreted as descriptive evidence of task-context-related topographic organization rather than as definitive evidence for independent task-specific microstate classes or as a strict common-template test of condition effects.

By contrast, group-related differences between DLD and TD were limited in conventional temporal microstate features. Conventional microstate parameters did not show robust corrected group effects or group × condition interactions under the present sample size, data-quality criteria, and multiple-comparison framework. This should not be interpreted as evidence that neural dynamics are unaffected in DLD. Rather, the findings indicate that small-to-moderate, state-specific, or clinically heterogeneous DLD-related effects may not have been reliably captured by the present conventional microstate measures.

Transition-based analyses also showed task-context-related variation in switching organization, but only sparse exploratory evidence for pathway-specific between-group differences. No Δ-transition pathway survived correction across the full testing family; therefore, the transition results should be regarded as hypothesis-generating rather than as evidence for a robust DLD-related transition abnormality.

These findings indicate that task context is a major determinant of preschool microstate dynamics and support the value of examining both conventional microstate features and transition structure in studies of early language-related neurodevelopmental differences. At the same time, the present findings place important constraints on biomarker claims: conventional EEG microstate parameters, as applied here, do not support standalone diagnostic differentiation of preschool DLD. Larger studies with more clinically characterized samples, predefined transition or syntax metrics, and pooled group-level or meta-microstate approaches are needed to determine whether EEG microstate features can reliably capture clinically meaningful DLD-related neural alterations.

## Data Availability

The datasets analyzed in this study are not publicly available due to ethical and privacy considerations related to the protection of the participating children’s anonymity. Requests to access the de-identified datasets should be directed to the corresponding authors and will be considered in accordance with the original ethical approval and applicable institutional requirements.
