# Supplementary material for: EEG microstate reconfiguration across cognitive states in preschool children with developmental language disorder
Source: Front Neurosci. 2026 Aug 17;20:1844934. doi: 10.3389/fnins.2026.1844934 (PMC13526948; doi:10.3389/fnins.2026.1844934)
Supplement: Supplementary file 1 [file Data_Sheet_1.pdf]

Supplementary Table S1. Rank-ordered model-order evaluation for EEG microstate clustering across conditions.

Panel A. Global ranking of candidate model orders across conditions

Global rank	Clusters (k)	RS rank	ODD rank	MAT rank	Mean recommended rank	Mean MetaCrit	Mean GEV (%)
**1**	**5**	**1**	**2**	**2**	**1.67**	**54.17**	**78.61**
2	6	3	3	1	2.33	50.00	79.91
3	7	4	1	3	2.67	33.33	80.17
4	4	2	4	3	3.00	25.00	75.72
5	1	4	4	3	3.67	0.00	61.73
5	2	4	4	3	3.67	0.00	66.22
5	3	4	4	3	3.67	0.00	71.66
5	8	4	4	3	3.67	0.00	81.95
5	9	4	4	3	3.67	0.00	82.40

Panel B. Condition-level rank-ordered model-order evaluation

Condition	Recommended rank	Clusters (k)	GEV (%)	MetaCrit
**Rest (RS)**	**1**	**5**	**80.41**	**100.00**
Rest (RS)	2	4	77.41	75.00
Rest (RS)	3	6	81.51	25.00
Rest (RS)	4	1	61.03	0.00
Rest (RS)	4	2	66.42	0.00
Rest (RS)	4	3	73.08	0.00
Rest (RS)	4	7	83.00	0.00
Rest (RS)	4	8	84.57	0.00
Rest (RS)	4	9	85.19	0.00
Oddball (ODD)	1	7	82.19	100.00
**Oddball (ODD)**	**2**	**5**	**80.55**	**50.00**
Oddball (ODD)	3	6	83.29	25.00
Oddball (ODD)	4	1	60.32	0.00
Oddball (ODD)	4	2	67.24	0.00
Oddball (ODD)	4	3	73.26	0.00
Oddball (ODD)	4	4	77.50	0.00
Oddball (ODD)	4	8	83.84	0.00
Oddball (ODD)	4	9	83.48	0.00
Match (MAT)	1	6	74.92	100.00
**Match (MAT)**	**2**	**5**	**74.86**	**12.50**
Match (MAT)	3	1	63.84	0.00
Match (MAT)	3	2	64.99	0.00
Match (MAT)	3	3	68.63	0.00
Match (MAT)	3	4	72.26	0.00
Match (MAT)	3	7	75.33	0.00
Match (MAT)	3	8	77.43	0.00
Match (MAT)	3	9	78.54	0.00

Note. RS = resting-state condition; ODD = passive auditory oddball condition; MAT = semantic picture-sound matching condition; GEV = global explained variance. Lower recommended-rank values indicate stronger model-order support within a condition. Panel A summarizes the candidate model orders across the three conditions by averaging the condition-level recommended ranks; mean MetaCrit and mean GEV are provided as descriptive indices. The five-cluster solution showed the best overall cross-condition ranking

and was therefore retained for the main microstate analyses. Because GEV tends to increase with larger k values, it was considered alongside MetaCrit and cross-condition stability rather than used as the sole selection criterion.

Supplementary Table S2. Exploratory group comparisons of Δ-transition indices across the three condition-contrast families.

Contrast	Transition	TD n	DLD n	TD mean ± SD	DLD mean ± SD	Mean difference (DLD - TD)	t	df	p	q (FDR)	Hedges' g	FDR significant
MR	C→B	22	12	-0.094 ± 0.049	-0.126 ± 0.075	-0.033	-1.363	16.385	0.191	0.984	-0.538	No
MR	E→D	22	12	-0.085 ± 0.064	-0.110 ± 0.052	-0.025	-1.244	27.160	0.224	0.984	-0.409	No
MR	E→B	22	12	-0.063 ± 0.073	-0.088 ± 0.114	-0.026	-0.700	16.060	0.494	0.984	-0.279	No
MR	C→E	22	12	-0.165 ± 0.061	-0.151 ± 0.069	0.015	0.625	20.531	0.539	0.984	0.227	No
MR	B→D	22	12	-0.084 ± 0.075	-0.100 ± 0.103	-0.016	-0.477	17.491	0.639	0.984	-0.184	No
MR	E→C	22	12	-0.253 ± 0.050	-0.264 ± 0.093	-0.011	-0.380	14.596	0.709	0.984	-0.158	No
MR	D→B	22	12	-0.067 ± 0.051	-0.072 ± 0.043	-0.005	-0.279	26.334	0.782	0.984	-0.093	No
MR	C→D	22	12	-0.117 ± 0.067	-0.124 ± 0.106	-0.007	-0.202	15.882	0.843	0.984	-0.081	No
MR	D→E	22	12	-0.114 ± 0.056	-0.112 ± 0.037	0.002	0.129	30.431	0.899	0.984	0.040	No
MR	B→C	22	12	-0.250 ± 0.062	-0.253 ± 0.075	-0.003	-0.128	19.445	0.899	0.984	-0.048	No
MR	D→C	22	12	-0.239 ± 0.049	-0.237 ± 0.086	0.002	0.090	15.041	0.930	0.984	0.037	No
MR	B→E	22	12	-0.125 ± 0.069	-0.124 ± 0.082	0.001	0.020	19.512	0.984	0.984	0.007	No
OM	E→D	22	12	0.003 ± 0.020	0.036 ± 0.045	0.033	2.382	13.323	0.033	0.393	1.030	No
OM	D→E	22	12	0.060 ± 0.040	0.077 ± 0.051	0.018	1.029	18.533	0.317	0.877	0.388	No
OM	D→F	22	12	-0.023 ± 0.028	-0.016 ± 0.033	0.007	0.587	19.830	0.564	0.877	0.216	No
OM	E→C	22	12	0.049 ± 0.029	0.058 ± 0.048	0.009	0.575	15.478	0.574	0.877	0.232	No
OM	D→C	22	12	0.021 ± 0.018	0.026 ± 0.032	0.005	0.536	15.097	0.600	0.877	0.219	No

Contrast	Transition	TD n	DLD n	TD mean ± SD	DLD mean ± SD	Mean difference (DLD - TD)	t	df	p	q (FDR)	Hedges' g	FDR significant
OM	F→D	22	12	0.008 ± 0.027	0.003 ± 0.024	-0.005	-0.530	25.107	0.600	0.877	-0.180	No
OM	C→D	22	12	0.004 ± 0.022	0.009 ± 0.028	0.005	0.520	18.216	0.610	0.877	0.197	No
OM	C→F	22	12	-0.004 ± 0.024	0.000 ± 0.031	0.005	0.431	18.395	0.672	0.877	0.163	No
OM	E→F	22	12	-0.010 ± 0.034	-0.005 ± 0.048	0.005	0.338	17.221	0.739	0.877	0.131	No
OM	F→E	22	12	0.069 ± 0.047	0.075 ± 0.054	0.006	0.333	20.037	0.743	0.877	0.122	No
OM	C→E	22	12	0.088 ± 0.045	0.092 ± 0.055	0.004	0.194	19.295	0.848	0.877	0.072	No
OM	F→C	22	12	0.033 ± 0.025	0.035 ± 0.028	0.002	0.157	20.787	0.877	0.877	0.057	No
OR	C→E	22	12	-0.077 ± 0.052	-0.059 ± 0.026	0.018	1.379	31.784	0.177	0.900	0.401	No
OR	D→E	22	12	-0.054 ± 0.051	-0.034 ± 0.039	0.020	1.255	28.135	0.220	0.900	0.407	No
OR	E→A	22	12	-0.109 ± 0.048	-0.088 ± 0.059	0.021	1.073	18.943	0.297	0.900	0.401	No
OR	A→C	22	12	-0.236 ± 0.060	-0.208 ± 0.077	0.027	1.066	18.423	0.300	0.900	0.403	No
OR	C→A	22	12	-0.140 ± 0.052	-0.126 ± 0.067	0.015	0.647	18.372	0.526	0.986	0.244	No
OR	A→E	22	12	-0.054 ± 0.049	-0.044 ± 0.039	0.010	0.635	27.135	0.531	0.986	0.209	No
OR	D→C	22	12	-0.218 ± 0.051	-0.210 ± 0.063	0.008	0.366	18.877	0.718	0.986	0.137	No
OR	E→D	22	12	-0.081 ± 0.066	-0.074 ± 0.081	0.007	0.263	19.139	0.795	0.986	0.098	No
OR	D→A	22	12	-0.118 ± 0.046	-0.116 ± 0.044	0.002	0.139	23.868	0.891	0.986	0.048	No
OR	E→C	22	12	-0.204 ± 0.061	-0.206 ± 0.061	-0.002	-0.102	22.720	0.919	0.986	-0.036	No
OR	C→D	22	12	-0.113 ± 0.068	-0.115 ± 0.084	-0.002	-0.069	18.891	0.946	0.986	-0.026	No
OR	A→D	22	12	-0.095 ± 0.063	-0.095 ± 0.090	-0.001	-0.017	16.922	0.986	0.986	-0.007	No

Note. MR = Match minus Rest; OM = Oddball minus Match; OR = Oddball minus Rest; TD = typically developing; DLD = developmental language disorder; SD = standard deviation; df = degrees of freedom; FDR = false-discovery rate. Mean difference is calculated as DLD - TD. The p values are uncorrected Welch's t-test results; q values are Benjamini-Hochberg FDR-corrected values separately within each contrast-specific family of 12 pathways. No listed comparison survived FDR correction.

Supplementary Table S3. Group-level clinical and language characteristics of the DLD group included in the final EEG microstate analyses

**Clinical/language characteristic**	**Assessment/source**	**DLD group, n/N (%) or summary**	**Notes**
Final analyzable DLD sample	EEG quality control for the present microstate analyses	12/12 (100.0%)	Final analyzable sample for the current study.
Nonverbal IQ/DQ > 70	WISC-IV or CNBS	12/12 (100.0%)	All included children met the nonverbal cognitive/developmental inclusion criterion.
Clinically confirmed persistent language-development difficulty	Standardized Mandarin language assessment, clinical speech-language evaluation, and caregiver interview records	12/12 (100.0%)	All included DLD children had language-development difficulty or delay documented for at least one year.
Articulation-related clinical feature	Standardized articulation assessment, oromotor function examination, clinical medical records, and caregiver behavioral reports	3/12 (25.0%)	Reported as an associated clinical feature, not necessarily as a separate formal diagnosis.
Speech-fluency-related clinical feature	Clinical speech-fluency assessment, medical records, and/or caregiver reports	3/12 (25.0%)	Reported as a fluency-related concern, including stuttering-like symptoms where documented.
Social-communication-related concern	Standardized social-communication evaluation, medical records, and/or caregiver reports	1/12 (8.3%)	Not equivalent to autism spectrum disorder unless formally diagnosed.
Attention-related concern	Standardized attention/behavior rating scales, medical records, and/or caregiver reports	3/12 (25.0%)	Not equivalent to ADHD unless formally diagnosed.
No additional associated clinical feature recorded beyond language difficulty	Comprehensive review of available medical charts and caregiver developmental interviews	2/12 (16.7%)	Based on available records; this category is mutually exclusive with the associated-feature rows above.
Speech-language therapy status	Rehabilitation medical records and/or caregiver follow-up reports	Receiving: 8/12 (66.7%); not receiving: 2/12 (16.7%); not systematically recorded / not available: 2/12 (16.7%)	Detailed information on intervention type, duration, and intensity was not systematically recorded / not available.

Note. Values are presented as n/N (%). DLD = developmental language disorder; IQ = intelligence quotient; DQ = developmental quotient; WISC-IV = Wechsler Intelligence Scale for Children-Fourth Edition; CNBS = Children Neuropsychological and Behavioral Scale; ADHD = attention-deficit/hyperactivity disorder. Clinical features were summarized from available clinical records, caregiver reports, and standardized developmental/language assessments. Categories are not necessarily mutually exclusive unless otherwise stated. Associated clinical features, such as attention-related or social-communication-related concerns, should not be interpreted as formal comorbid diagnoses unless clinically confirmed. Individual-level raw assessment scores and detailed clinical records are not publicly reported because the participants were preschool children and these data involve ethical and privacy restrictions.
